# Molybdenum–Tungsten Blue Nanoparticles as a Precursor for Ultrafine Binary Carbides

**DOI:** 10.3390/nano11030761

**Published:** 2021-03-17

**Authors:** Maria Myachina, Natalia Gavrilova, Ksenia Poluboyarinova, Victor Nazarov

**Affiliations:** Department of Colloid Chemistry, D. Mendeleev University of Chemical Technology of Russia, Miusskaya sq., 9, 125047 Moscow, Russia; ngavrilova@muctr.ru (N.G.); amalieleon@gmail.com (K.P.); nazarov@muctr.ru (V.N.)

**Keywords:** molybdenum–tungsten blue, polyoxometalate complex, sol–gel method, binary carbide, transition metal carbide

## Abstract

Herein, we demonstrate a promising method for the synthesis of ultrafine carbide particles using dispersions of molybdenum–tungsten nanoparticles. Dispersions of molybdenum–tungsten blue nanoparticles with different initial molar ratios of molybdenum/tungsten were synthesized through the reduction of molybdate and tungstate ions by ascorbic acid in an acidic medium (pH = 1.0–2.5). Molybdenum–tungsten blue nanoparticles were characterized by ultraviolet–visual (UV–VIS), infrared (FTIR), and X-ray photoelectron (XPS) spectroscopies; transmission electronic microscopy (TEM); and dynamic light scattering (DLS). We demonstrated that molybdenum–tungsten blue nanoparticles belong to toroidal polyoxometalate clusters (λ_max_ = 680–750 nm) with a predominant particle size of 4.0 nm. Molybdenum–tungsten blue dispersions were shown to be monodispersed systems with a small particle size and long-term stability (>30 days) and are suitable for further catalytic applications.

## 1. Introduction

The use of highly dispersed molybdenum and tungsten carbides is one of the promising directions in the development of catalytic processes, including hydrogen evolution reactions and the production of synthesis gas [1,2,3,4,5,6]. The main trend in the preparation of molybdenum and tungsten carbides is the focus on the synthesis of highly dispersed catalytic material. However, due to the fact that transition metal carbides are formed at high temperatures, it is difficult to obtain molybdenum or tungsten carbide particles with a small size and highly specific surface area [7,8,9,10].

The temperature-programmed reduction (TPR) of molybdenum–tungsten carbide precursors (MoO_3_, WO_3_, MoO_2_, WO_2_, etc.) in the medium of various carburizing agents (CH_4_/H_2_, C_2_H_6_/H_2_, C_4_H_10_/H_2_, etc.) is the most common method for synthesizing a highly dispersed material [11]. The formation of molybdenum or tungsten carbide particles with a size not exceeding tens of micrometers is realized through the use of a highly dispersed precursor. The main disadvantage of this method is the presence of a long stage of carburization, which lasts from one to several hours depending on the process temperature. As a result of TPR, an additional carbon layer may form on the surface of the carbide, which requires preliminary passivation of the catalytic material [12].

In addition to temperature-programmed carburization in a gas medium, researchers proposed using a liquid as a carbon source, for example, urea [13] or various carbon materials [14,15]. Thus, either the liquid-phase or solid-phase synthesis of molybdenum carbide is realized. The main requirement for the sol–gel method is the ability to obtain highly dispersed carbides. Thus, a sol–gel method that utilizes a disperse system of molybdenum–tungsten blue is preferred.

Nanoparticles of molybdenum–tungsten blues can contain polyoxometalate (POM) complexes or nanoclusters containing molybdenum and tungsten in variable oxidation states. Polyoxometalate complexes have a fixed size of the order of 3–5 nm [16,17,18,19,20,21]. Today, the class of polyoxometalate complexes is being actively studied, and structures of various compositions, shapes, and sizes were previously obtained [22,23,24,25,26]. Research found that polyoxometalates containing molybdenum are formed as a result of the self-assembly process from the initial building blocks Mo_1_, Mo_2_, Mo_8_, etc. [17,27].

Interest in polyoxometalate complexes, including molybdenum blue and tungsten blue nanoparticles, is due to their unique physicochemical properties, structural lability, and high reactivity. The most promising areas of their application are the creation of hybrid materials, targeted drug delivery systems, nanoreactors, and catalytic materials [28,29]. There is insufficient information regarding the properties of dispersions of tungsten blue—the most important of which is the low stability of dispersions of tungsten blue [30]. Tungsten blues are strong reducing agents and their aqueous dispersions cannot be stored in air (they are rapidly oxidized to tungsten trioxide). To ensure their chemical stability, the introduction of additional stabilizers is required.

Even though POM containing tungsten mainly exists in the form of heteropoly compounds containing other elements in water systems or in the form of polyoxotungstates in organic solvents, data on the synthesis of binary molybdenum–tungsten blue are limited in the literature [31,32,33].

The use of a highly dispersed precursor, containing polyoxometalate nanoclusters, leads to the formation of carbide phase with a small particle size and high specific surface area [28,34]. However, the first stage in the development of a method for obtaining highly dispersed carbides is to establish the conditions for the formation of molybdenum–tungsten blue nanoparticles and the possibility of obtaining their stable dispersions.

The choice of conditions for the synthesis of stable dispersions of molybdenum–tungsten blue and the analysis of the size and particle structures were carried out for the first time in this work. The significance of this study was confirmed by the results already obtained on the synthesis of binary molybdenum and tungsten carbides. In our previous work [35], we demonstrated that the use of nanoparticles of molybdenum–tungsten blue made it possible to obtain a highly dispersed catalytic material with a microporous structure and a high specific surface area of approximately 150 m^2^/g.

The aim of this work is to develop favorable conditions for the synthesis of dispersions of molybdenum–tungsten blue and to determine the main properties (size and structure) of molybdenum–tungsten blue nanoparticles and their dispersion properties (stability time and particle concentration).

## 2. Materials and Methods

### 2.1. Materials

Molybdenum–tungsten blue dispersions were synthesized using the following reagents: ammonium heptamolybdate ((NH_4_)_6_Mo_7_O_24_∙4H_2_O), ammonium tungstate ((NH_4_)_10_W_12_O_41_ · 5H_2_O,), crystalline ascorbic acid (C_6_H_8_O_6_), and hydrochloric acid (HCl). All reagents were of reagent grade and delivered by CT Lantan (Moscow, Russia).

### 2.2. Synthesis of Molybdenum–Tungsten Blue Dispersions

Dispersions of molybdenum–tungsten blue were obtained as a result of the reduction of molybdate and tungstate solution by ascorbic acid. The synthesis was carried out at the constant metal concentration 0.07 M (the sum of molybdenum and tungsten), at the range of molar ratio (Mo)/(W) = 50/50–100/0 and constant molar ratios (H)/(Me) = 0.6 and (R)/(ΣMe) = 1.0. The total particle concentration (% wt. MoO_3_–WO_3_) was determined by calcinating samples of molybdenum–tungsten blue in the air at 600 °C.

### 2.3. Characterization of Molybdenum–Tungsten Blue Dispersions

UV–VIS spectra were recorded using a Leki SS2110 UV scanning spectrophotometer (MEDIORA OY, Helsinki, Finland) using quartz cells. The absorbance measured at the absorption maximum was used in linear correlation with the relative particle concentration according to the Beer–Lambert law. The hydrodynamic diameter of the particles in the molybdenum–tungsten blue dispersions were determined by dynamic light scattering using a Photocor Compact-Z analyzer (OOO Photocor, Moscow, Russia). The signal accumulation lasted 30 min at a laser power of 20 mW and a wavelength of 658 nm.

The sizes of the particles were determined with a LEO 912AM Omega (Carl Zeiss, Jena, Germany) transmission electron microscope. Images were acquired at a 100 kV accelerating voltage. Analysis of the microphotographs and calculation of particle sizes were carried out using the Image Tool V.3.00 (Image Tool Software, UTHSCSA, San Antonio, CA, USA). FTIR spectra were measured using a Nicolet 380 IR Fourier spectrometer (Thermo Fisher Scientific Inc., Waltham, MA, USA) in compressed KBr pellets in the range of 350 to 4000 cm^−1^. The XPS spectra were recorded on an ESCA + X-ray photoelectron spectrometer (OMICRON Nanotechnology GmbH, Taunusstein, Germany).

## 3. Results

Self-assembly of molybdenum blue nanoclusters occurs during the reduction of solutions of molybdate ions in an acidic medium [18,19,20]. We supposed that dispersions of molybdenum–tungsten blue would be formed as a result of molybdate and tungstate ion reduction. Ascorbic acid was chosen as a reducing agent because other types of reducing agent, such as glucose or hydroquinone, have insufficient reducing power to reduce tungstate, according to the literature [30].

### 3.1. Characterization of Molybdenum–Tungsten Blue Nanoparticles

At the first stage, it was necessary to establish an optimal content of the reducing agent in the system. According to the synthesis of molybdenum blue dispersions using ascorbic acid, the optimal molar ratio was (R)/(Mo) from 0.8 to 1 [21]. In this work, we established that stable molybdenum–tungsten blue dispersions could also be obtained within the same range of the molar ratio (R)/(Me) and at the molar ratio (H)/(Me) = 0.6. The molar ratios (R)/(Me) = 1 and (H)/(Me) = 0.6 were used for further synthesis of dispersions and their investigation.

**Figure 1:**
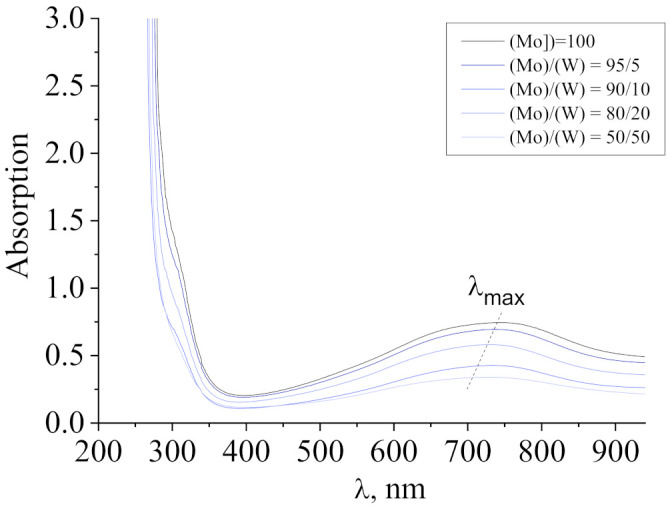
The electronic absorption spectrum of dispersion of molybdenum–tungsten blue synthesized using ascorbic acid ((R)/(Mo) = 1) and with different molar ratios (Mo)/(W).

Figure 1 shows the absorption spectra of the molybdenum–tungsten blue dispersions synthesized at the molar ratio (R)/(Me) = 1 and molybdenum–tungsten molar ratios (Mo)/(W) = 95/5; 90/10; 80/20; and 50/50. The spectrum of the molybdenum blue dispersion ((Mo) = 100, without W) was used as a benchmark.

As can be seen, with an increase in the tungsten content, the absorption maximum shifted from 750 to 680 nm, which caused the color change of the dispersions from dark blue to blue with a violet tone. This shift may be associated with the possible incorporation of tungsten compounds into the structure of the molybdenum oxide nanoclusters [21]. Dispersions of molybdenum blue synthesized using ascorbic acid were characterized by the presence of an absorption maximum at 745 nm. This value λ_max_ is characteristic of toroidal molybdenum oxide nanoclusters of the Mo_154-x_ family [25,26].

In general, the change in the absorption spectrum with an increase in the tungsten content in the system can occur for several reasons:

- the resulting polyoxometalate complexes differ in composition and structure from molybdenum oxide nanoclusters of the Mo_154-x_ family;
- molybdenum oxide nanoclusters of the Mo_154-x_ family are formed in the system, on the surface of which tungsten ions are adsorbed in a variable oxidation state; and
- the dispersed phase is represented by Mo_154-x_ nanoclusters, and the system contains dissolved colored reduced forms of tungsten.

**Figure 2:**
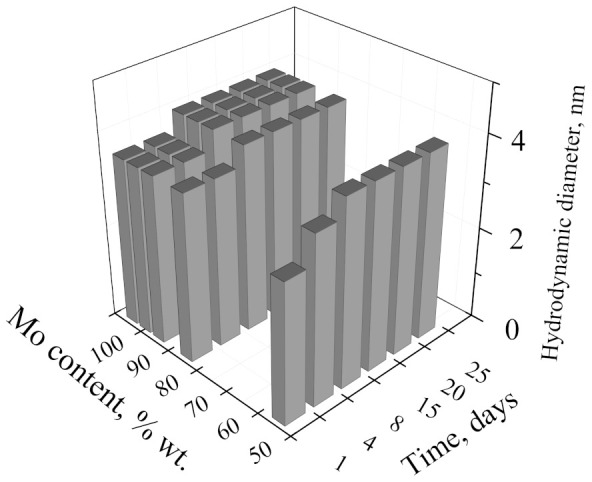
The dependence of the hydrodynamic diameter of particles on the composition of molybdenum–tungsten blue particles (molar ratio (R)/(Me) = 1.0; (H)/(Me) = 0.6).

In this regard, dynamic light scattering (DLS) data provide additional information regarding the formation of the molybdenum–tungsten blue particles. According to this data, the formation of molybdenum–tungsten blue particles occurs during one day after mixing all reagents. Figure 2 shows the results obtained for the samples with a molar ratio (H)/(Me) = 0.6. A similar dependence was observed for all investigated molar ratios (H)/(Me). Over time, the value of the hydrodynamic diameter did not change and was in the order of 4.0 nm. This value of the diameter is comparable with the characteristic size of toroidal clusters in molybdenum blues [19,20,22].

According to the DLS data, only one dominant particle diameter was observed, which suggests the formation of particles of a binary compound of molybdenum and tungsten.

**Figure 3:**
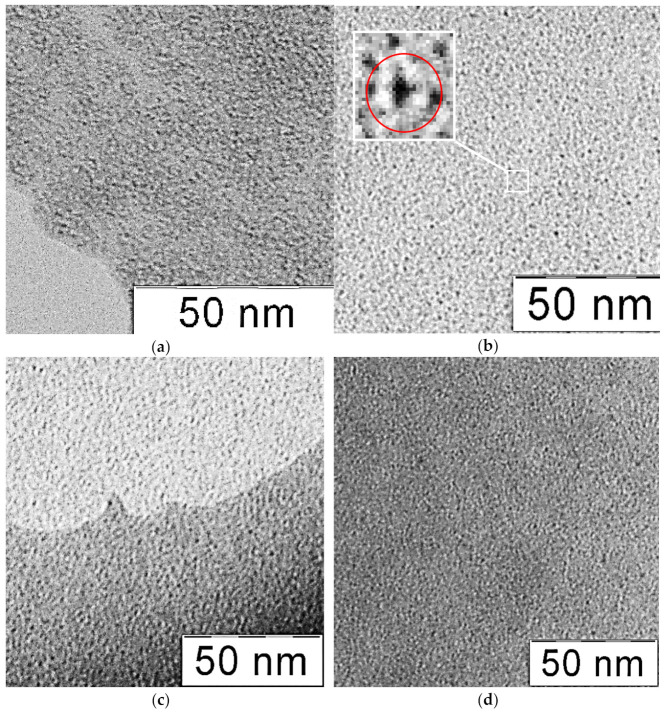
Transmission electron microscopy (TEM) images of molybdenum–tungsten blue nanoparticles, synthesized with various molar ratios: (Mo)/(W) = 95/5 (**a**), (Mo)/(W) = 90/10 (**b**), (Mo)/(W) = 80/20 (**c**), and (Mo)/(W) = 50/50 (**d**).

The sizes and shapes of the particles were analyzed using transmission electron microscopy (TEM). Figure 3 shows TEM micrographs of molybdenum–tungsten nanoparticles.

The micrographs show that the particles of molybdenum–tungsten blue were toroidal particles with a fixed size. The estimation of the particle size showed that the diameter of the tori was in the order of 3–4 nm. However, a more accurate estimation of the size was impossible due to reaching the limits of the resolution. The size distribution and micrographs were similar for all investigated samples with different initial molar ratios (Mo)/(W). The TEM images for molybdenum oxide nanoclusters obtained by reduction of molybdates with ascorbic acid were very close to these results [21]. This confirms our assumption that the properties of nanoparticles of molybdenum blue and molybdenum–tungsten blue are very similar.

**Figure 4:**
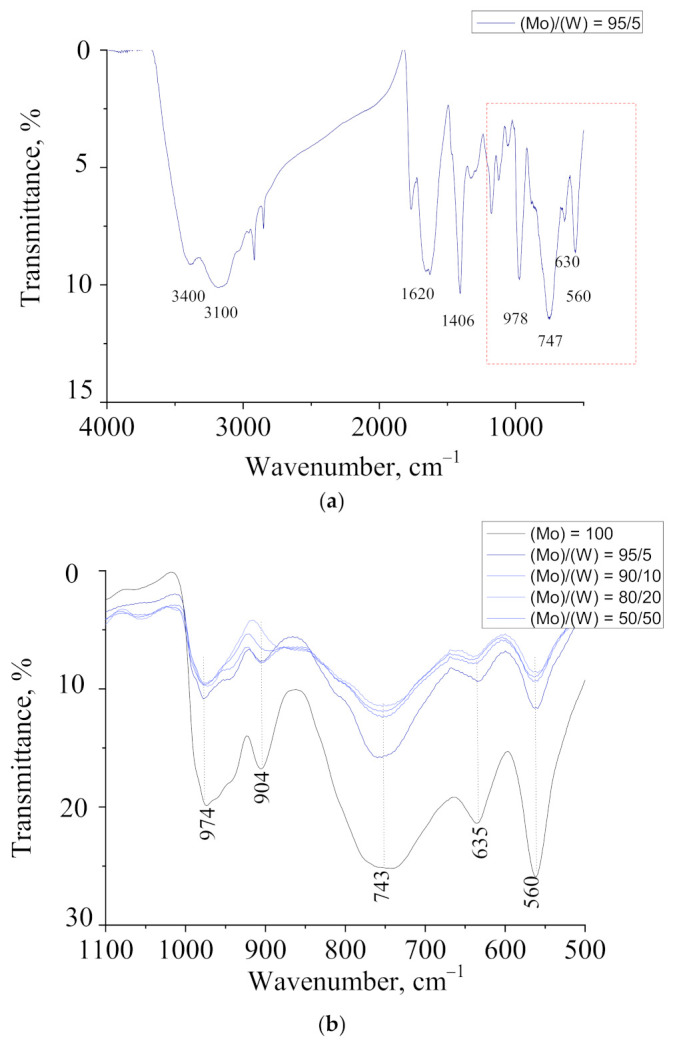
FTIR spectra of molybdenum–tungsten oxide nanoclusters with different molar ratios (Mo)/(W) isolated from the dispersions synthesized: (**a**) general spectrum (500–4000 cm^−1^) for sample (Mo)/(W) = 95/5 and (**b**) spectra (500–1100 cm^−1^).

FTIR spectroscopy was used to determine the structure of the synthesized molybdenum–tungsten blue nanoparticles. Figure 4 shows the FTIR spectra for samples with different molar ratios (Mo)/(W) = 95/5; 90/10; 80/20; and 50/50, and, for comparison, the spectrum of pure molybdenum blue nanoparticles ((Mo) = 100) was used. The assignment of bands is presented in Table 1.

In the region of 1000–500 cm^−1^, there were bands that characterize polyoxometalate structures, including molybdenum oxide nanoclusters. For all samples of molybdenum–tungsten blues obtained with the molar ratio of the initial precursors (Mo)/(W) = 95/5; 90/10; 80/20; and 50/50, bands at 977, 902, 737, 634, and 561 cm^−1^ were observed, corresponding to the structure of toroidal complexes of the cluster family Mo_154-x_ [22,25]. Bands characterizing tungsten–oxygen bonds (W = O and W–O–W) were not found [36]. Considering these results, we assumed that tungsten was incorporated into the structure of toroidal molybdenum oxide nanoclusters.

The particles demonstrated the same high hydration as molybdenum blue, as evidenced by the bands corresponding to hydrogen bonds ν(OH…H) and bending vibrations of water molecules δH_2_O. The spectra of molybdenum–tungsten blue nanoclusters, obtained at different precursor molar ratios (Mo)/(W), were similar to the spectrum of molybdenum blue, especially the toroidal molybdenum oxide nanoclusters [21].

**Figure 5:**
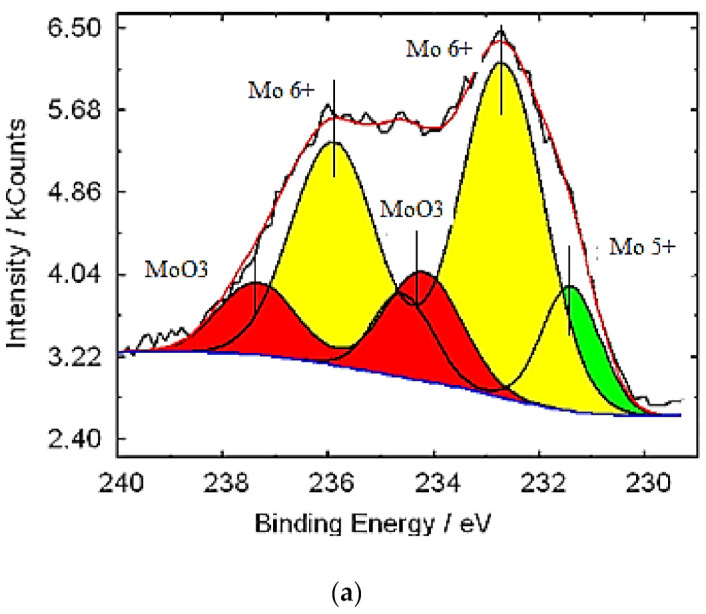

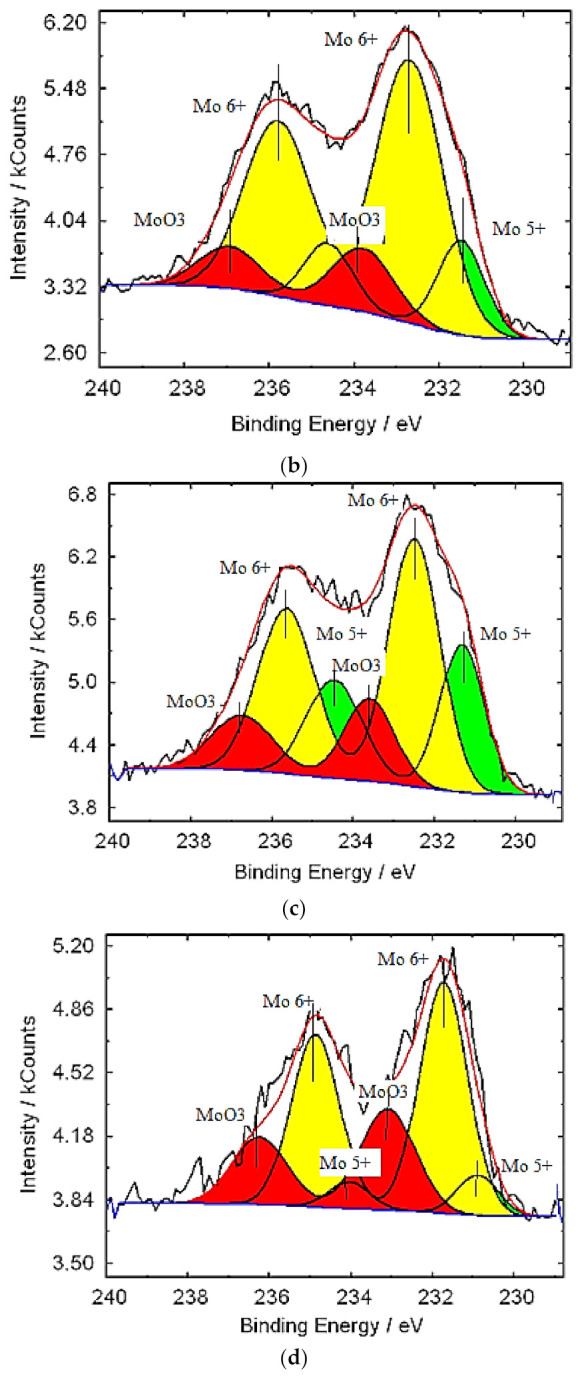
X-ray photoelectron spectroscopy (XPS) spectrum of Mo (**a**–**d**) in molybdenum–tungsten oxide nanoclusters: (Mo)/(W) = 95/5 (**a**); (Mo)/(W) = 90/10 (**b**); (Mo)/(W) = 80/20 (**c**); and (Mo)/(W) = 50/50 (**d**).

**Figure 6:**
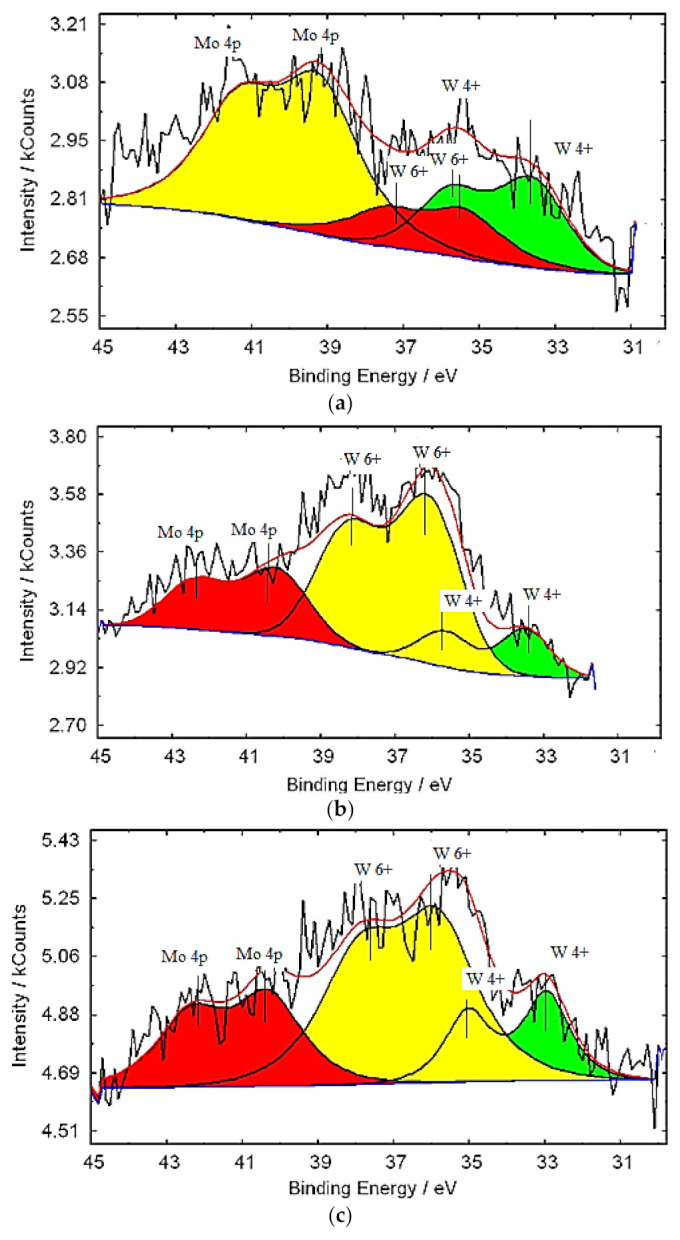

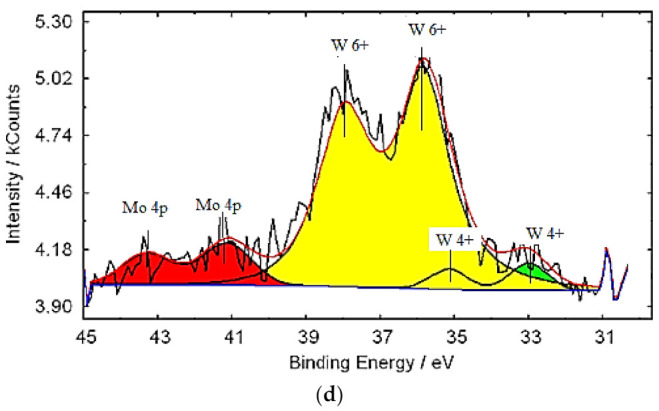
X-ray photoelectron spectroscopy (XPS) spectra of W (**a**–**d**) in molybdenum–tungsten oxide nanoclusters: (Mo)/(W) = 95/5 (**a**); (Mo)/(W) = 90/10 (**b**); (Mo)/(W) = 80/20 (**c**); and (Mo)/(W) = 50/50 (**d**).

X-ray photoelectron spectroscopy (XPS) was used to establish the oxidation state of molybdenum and tungsten in molybdenum–tungsten blue nanoparticles. The lines of internal electrons were used. For molybdenum, this is the region of the line of Mo3d electrons and W4f for tungsten. Figure 5 and Figure 6 depict the results of the interpretation of the XPS spectra for molybdenum–tungsten blue nanoparticles of various compositions.

The spectrum of Mo (Figure 5) is represented by three doublets: Mo^6+^ (electrons 3d5/2 and 3d3/2 with binding energies of 232 and 237 eV), Mo^5+^ d5/2 and Mo^5+^ d3/2 (electrons d5/2 and d3/2 with binding energies of 231 and 235 eV), and Mo^6+^ (MoO_3_) (electrons with binding energies of 234.6 and 237.7 eV). The tungsten spectrum is described by two doublets: W^6+^ (electrons 4f5/2 and 4f7/2 with binding energies of 37.8 and 35.7 eV) and W^4+^ (electrons 4f5/2 and 4f7/2 with binding energies of 33.8 and 35.7 eV).

The tungsten spectrum (Figure 6) is described by two doublets: W^6+^ (electrons 4f5/2 and 4f7/2 with binding energies of 37.8 and 35.7 eV) and W^4+^ (electrons 4f5/2 and 4f7/2 with binding energies of 33.8 and 35.7 eV).

Thus, in the composition of molybdenum–tungsten clusters, molybdenum exists in the form of Mo^V^ and Mo^VI^ and tungsten as W^IV^ and W^VI^. The presence of tungsten with the oxidation state of +4 in the cluster composition likely leads to a change of the absorption spectra and a shift of the absorption maximum to a shorter wavelength region as shown earlier in Figure 1.

### 3.2. Properties of Molybdenum–Tungsten Blue Dispersions

**Figure 7:**
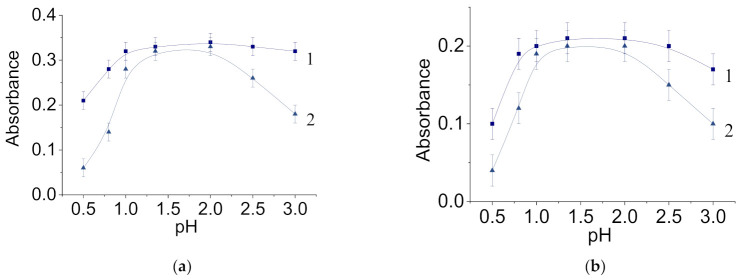

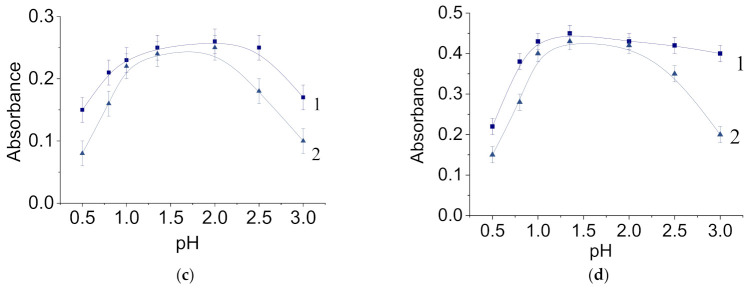
The dependence of the absorbance on the pH of the dispersion medium for dispersions of molybdenum–tungsten blue of various compositions: (Mo)/(W) = 95/5 (**a**), (Mo)/(W) = 90/10 (**b**), (Mo)/(W) = 80/20 (**c**), and (Mo)/(W) = 50/50 (**d**). The measurements were carried out 1 (curve 1) and 7 (curve 2) days after preparation of the samples.

For further application of molybdenum–tungsten blue as a precursor for highly dispersed molybdenum and tungsten carbides, it is necessary to establish some properties, including the pH range in which the dispersions retain their stability. Figure 7 shows the dependences of the absorbance measured at the absorption maximum on the pH of the dispersion medium of the systems.

As can be seen, for all the systems, a stable pH was reached where the absorbance was relatively constant for samples that were 1 day old. With increasing the storage time to 7 days, the pH declined after reaching the maximum values. These trends were observed for all investigated molar ratios (Mo)/(W).

The considered range of pH values at which the systems remain stable is also typical for dispersions of molybdenum blue. The stability of molybdenum blue dispersions in the pH range of 1.0 to 2.5 was due to the highest concentration of polymolybdate ions, which act as potential-determining ions for molybdenum oxide nanoclusters [38]. In the neutral pH region, the dissolution of polyoxometalate complexes occurs, while in the more acidic pH region, the dispersions lose their stability.

For further applications in sol–gel technology, it is necessary to establish for how long the dispersions of molybdenum–tungsten blue retain their chemical and aggregate stability. The stability of the systems was defined as the time during which there is no change in the concentration of particles, including due to the formation of a precipitate. In the case of molybdenum oxide nanoclusters, the UV–VIS spectroscopy can be used as a quantitative method of analysis [16,17]. Thus, for controlling the stability of dispersions, the time dependences of maximum absorbance (λ_max_ = 745 nm) were used.

The concentration of the dispersed phase is also an important parameter for the sol–gel technology as this determines the properties of dispersion and the final product. Table 2 shows the parameters for the dispersions obtained with the initial molar ratios of precursors (Mo)/(W) = 95/5; 90/10; 80/20; and 50/50.

As shown in Table 2, all synthesized dispersions remained stable for at least 1 month. The concentration of the dispersed phase for all systems was 1 wt.%, and there is the possibility of further increases in concentration up to 10 wt.%. The small particle size, long-term stability, and the possibility of obtaining dispersions with a given particle concentration make molybdenum–tungsten blue dispersions a promising precursor for preparation of catalytic materials via the sol–gel method [35].

## 4. Discussion

In this work, we discussed unique properties of nanoparticles of molybdenum–tungsten blue, synthesized as a result of the reduction of solutions of molybdate and tungstate ions with ascorbic acid. Previously, we studied, in detail, the formation of nanoparticles of molybdenum blue, synthesized using various organic reducing agents [21,38,39]. The developed synthesis method is suitable for obtaining stable dispersions of both molybdenum blue and molybdenum–tungsten blue and applicable for obtaining catalytic materials based on transition metal carbides with the sol–gel method [34,35,40,41]. However, the significance of the research carried out in the field of the synthesis of dispersions of molybdenum and molybdenum–tungsten blue lies not only in applied applications but also in considering the fundamental problems of formation-associated colloid systems [42,43].

Analyzing the obtained data on the properties of dispersions, we assumed that the dispersions of molybdenum and molybdenum–tungsten blues would have similar behavior to micellar surfactant solution-associated (lyophilic) dispersion systems. First, the formation of nanoparticles of molybdenum and molybdenum–tungsten blue (POM) occurs spontaneously as a result of self-assembly, as in the case of micelle formation. Second, nanoparticles of molybdenum and molybdenum–tungsten blue have a small particle size and can be attributed to monodisperse systems, similar to micellar surfactant solutions. Third, dispersions of molybdenum and molybdenum–tungsten blue under certain conditions can maintain their stability for a long time.

Consideration of the properties of dispersions of inorganic nanoparticles exhibiting the properties of lyophilic disperse systems, in particular, nanoparticles of molybdenum–tungsten blue, was performed for the first time. This study provides a better understanding of the process of self-assembly and the stability of inorganic lyophilic dispersed systems.

## 5. Conclusions

We demonstrated that molybdenum–tungsten blues are highly dispersed systems based on polyoxometalate complexes of molybdenum and tungsten. A unique property of POM is monodispersed particles with a particle diameter of 4 nm. The investigation of the effect of the molar ratio of reducing agent/metal (molybdenum and tungsten) and molar ratio of molybdenum/tungsten on the properties of dispersions showed that stable nanoparticles were formed at the molar ratio (R)/(Me) = 0.8–1 and at the initial molar ratios of molybdenum/tungsten (Mo)/(W) = 95/5; 90/10; 80/20; and 50/50.

According to the UV–VIS, FTIR, and XPS results, the structure of molybdenum–tungsten blue nanoparticles was very similar to the structure of molybdenum blue nanoparticles synthesized using ascorbic acid. This similarity can be explained from the position that the molybdenum–tungsten blue nanoparticles are giant mixed molybdenum–tungsten oxide nanoclusters. We investigated the structure and shape of molybdenum–tungsten blue nanoparticles for the first time. Our study of the properties of molybdenum–tungsten blue nanoparticles (mixed molybdenum–tungsten polyoxometalate complexes) contributes significantly to the development of soft-matter chemistry as it relates to the self-assembly of associated colloids.

The developed method for the synthesis of molybdenum–tungsten dispersions makes it possible to obtain a monodisperse system with a small particle size, long-term stability, and the possibility to control particle concentration. The studied systems were used for the synthesis of binary carbides of Mo_2_C–W_2_C using the sol–gel method [35]. Binary carbides of Mo_2_C–W_2_C obtained from molybdenum–tungsten blue nanoparticles are microporous and have a surface area of about 150 m^2^/g. The next step is to develop the sol–gel synthesis of membrane catalysts based on binary molybdenum and tungsten carbides. Establishing the relationship between the properties of dispersions of molybdenum–tungsten blue and the properties of the final product will make it possible to obtain membrane catalysts with specified parameters.

## Figures and Tables

**Table 1:** Assignment of several bands in the IR spectra of molybdenum–tungsten nanoparticles.

Band Position (cm^−1^)	Assignment	Reference Data
977 s 902 w	νMo=O	[37]
737 s 634 m	ν(Mo–μ_2_O–Mo) or ν(Mo–μ_3_O–Mo)	[37]
561 s	δ(O–Mo–O)	[37]
1620 s	δH_2_O	[37]
3400 s	ν(OH…H)	[37]
1407 w	δNH_4_^+^	[37]

s—strong, m—medium, w—weak, δ—bending vibrations, —stretching vibrations, and μ_2_O/μ_3_O —bridged oxygen atom connected with two or three molybdenum, respectively.

**Table 2:** Properties of molybdenum–tungsten nanoparticle dispersions ((R)/(Me) = 1.0; (H)/(Me) = 0.6), shown via dynamic light scattering (DLS).

Sample	Predominant Particle Size, nm (DLS)	Stability Time, Days	Particle Concentration, % wt. (MoO_3_–WO_3_)
(Mo)/(W) = 95/5	4.0	>30	1.0
(Mo)/(W) = 90/10	4.0	>30	1.0
(Mo)/(W) = 80/20	4.0	>30	1.0
(Mo)/(W) = 50/50	4.0	>30	1.0

## Data Availability

The data presented in this study are available on request from the corresponding author.
